# Supplementary figures and images for: Isolation and Characterization of a Novel Aeromonas salmonicida-Infecting Studiervirinae Bacteriophage, JELG-KS1
Source: Microorganisms. 2024 Mar 8;12(3):542. doi: 10.3390/microorganisms12030542 (PMC10974468; doi:10.3390/microorganisms12030542)

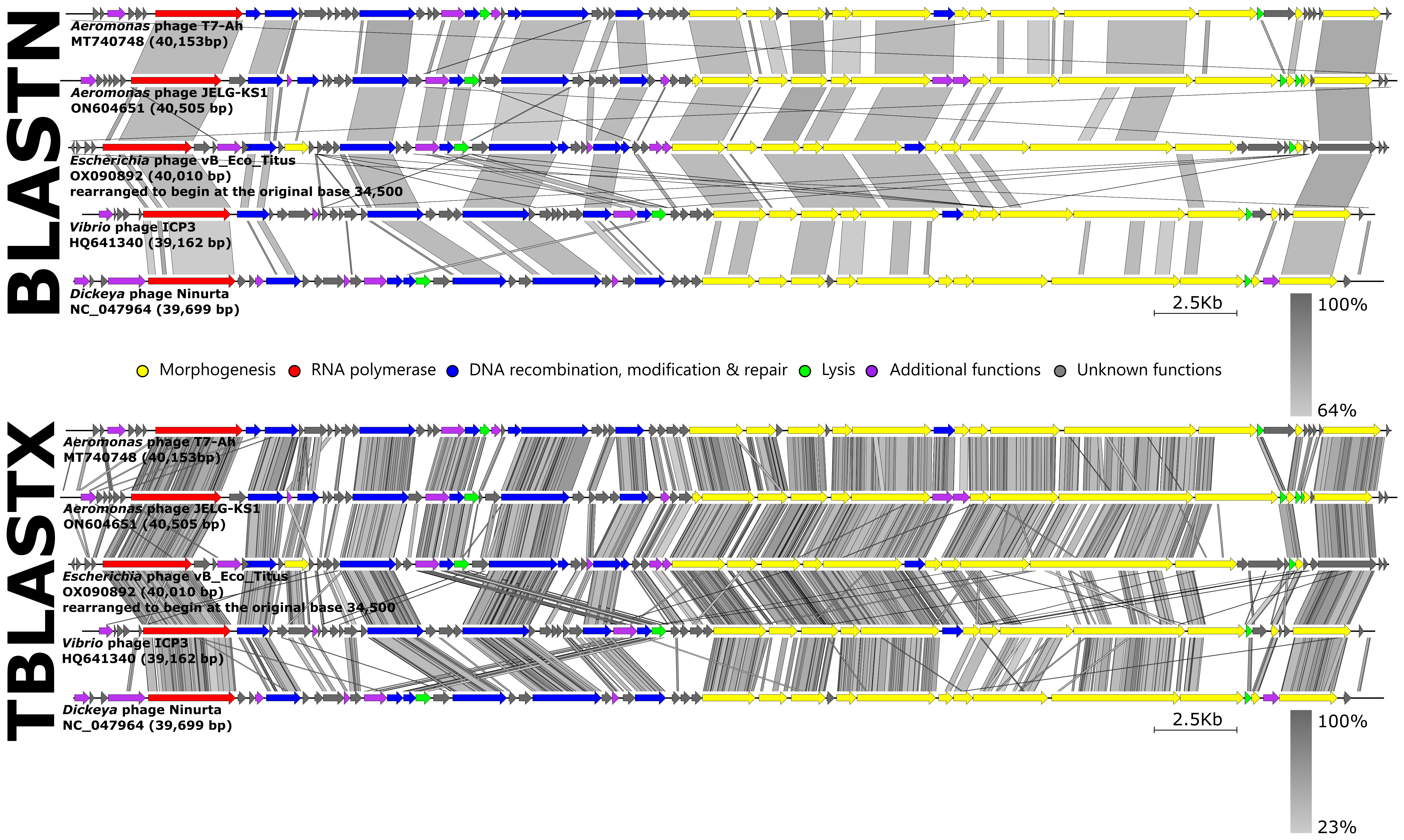

Supplement: Supplementary file 1 [file microorganisms-12-00542-s001.zip › FigS2.600dpi.png]

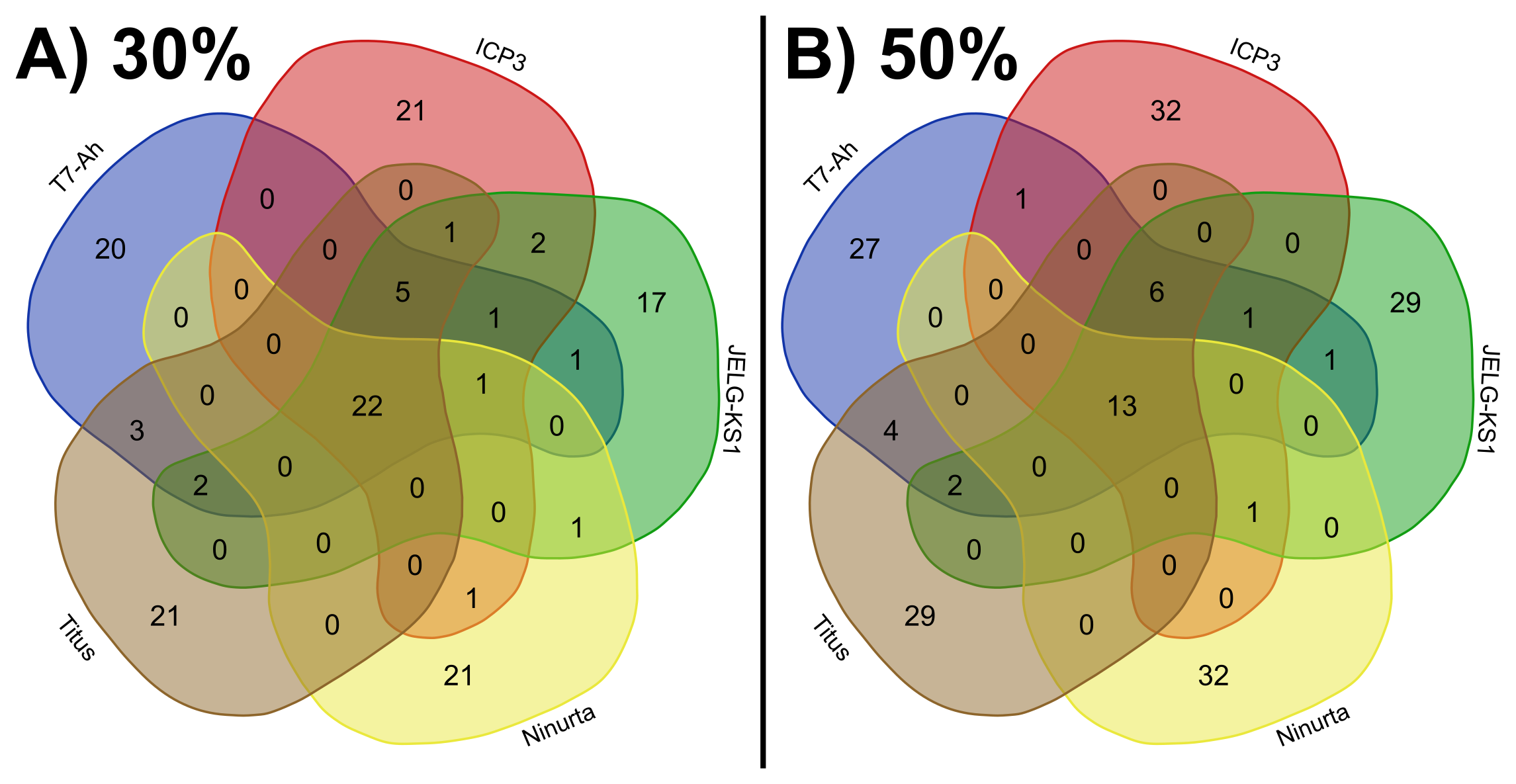

Supplement: Supplementary file 1 [file microorganisms-12-00542-s001.zip › FigS1.600dpi.png]
